# Using Photovoice Methods to Set Research Priorities With Autistic People With Experience of an Eating Disorder

**DOI:** 10.1002/jclp.23802

**Published:** 2025-05-03

**Authors:** Emy Nimbley, Kyle Buchan, Ellen Maloney, Sarah Kettley, Michelle Sader, Fiona Duffy, Karri Gillespie‐Smith

**Affiliations:** ^1^ School of Health in Social Science University of Edinburgh, Scotland Edinburgh UK; ^2^ Eating Disorder and Autism Collaborative (EDAC) University of Edinburgh, Scotland Edinburgh UK; ^3^ Edinburgh College of Art University of Edinburgh, Scotland Edinburgh UK; ^4^ School of Medicine, Medicinal Sciences and Nutrition University of Aberdeen Scotland; ^5^ NHS Lothian Child and Adolescent Mental Health Services Royal Edinburgh Hospital Scotland

**Keywords:** autism, eating disorders, photovoice

## Abstract

**Objective(s):**

Autism and Autistic traits are heightened in individuals with eating disorders (EDs), with Autistic people reporting poorer treatment outcomes and experiences. Despite this, mechanisms of this overlap remain poorly understood, perhaps due to an exclusion of lived experience perspectives in setting research agendas. The study therefore sought to identify research priorities for Autistic people with an eating disorder (ED) by using accessible and inclusive arts‐based research methodologies.

**Methods:**

Research questions were explored using Photovoice, a creative research methodology involving the creation and discussion of images. 14 participants attended group workshops, in two of which they explored research priorities for Autistic people with an ED. Images and transcripts were analysed using an adapted Photovoice Question Matrix (production, content and meaning of the image) and thematic analysis. Participants had the opportunity to provide feedback on emerging themes before themes were finalised.

**Results:**

Five themes were identified that highlighted research priorities for Autistic people with an ED: (1) Impact of early experiences (with subthemes I*nternalizing of socio‐cultural food and body narratives* and *generational cycles*); (2) Function of the ED (with sub‐themes *ED as a regulatory strategy* and *ED as a social acceptance strategy*); (3) Barriers and facilitators to ED recovery (with sub‐themes *Autistic traits as barriers, Autistic traits as facilitators* and *Help and harm of unravelling*); (4) Understanding and accommodating for complexity (with sub‐themes *Co‐occurring conditions* and *Intersectionality*); and (5) Changing research culture (with sub‐themes *Inclusive and participatory research* and *Nonclinical support*).

**Discussion:**

Study findings are contextualised within existing autism and ED research, highlighting avenues for future research and making recommendations for future research questions. By identifying community‐driven research priorities, it is hoped that study findings will inspire novel, interdisciplinary and co‐produced research that will serve as a meaningful evidence base towards improving the lives of Autistic people with an ED.

## 1 Introduction

Autism spectrum condition (ASC; here on referred to as autism, Kenny et al. 2016; Bottini et al. 2024) is characterised by differences in sensory processing, socio‐emotional communication and repetitive behaviours (American Psychiatric Association, D. S. M. T. F., & American Psychiatric Association, D. S 2013). Autistic individuals experience elevated rates of mental health difficulties (Lai et al. 2019), such as eating disorders (EDs; Adams et al. 2024). Autistic individuals report significant higher rates of ED symptoms than their non‐Autistic peers (Sedegwick et al. 2020), while between 25% and 28% of Autistic women report experience of an ED (Brown et al. 2024; Nuyttens et al. 2024). Furthermore, prevalence estimates of autism or elevated Autistic traits have been reported in ~30% of individuals with anorexia nervosa (AN; Westwood and Tchanturia 2017) with lower yet under‐researched estimates reported in binge eating disorder (BED), bulimia nervosa (BN) and avoidant and restrictive feeding intake disorder (ARFID) (Gesi et al. 2017; Sader et al. 2025). Autistic individuals may also have poorer treatment outcomes and experiences than non‐Autistic individuals (Nimbley et al. 2025) and may therefore require treatment adaptations Several conceptual models for the overlap between autism and EDs have been proposed, implicating a range of shared and autism‐specific social, emotional, sensory and cognitive factors, (Brede et al. 2020; Kinnaird and Tchanturia 2021; Carpita et al. 2022), however many of these mechanisms are still to be robustly empirically investigated, which may in turn be contributing to poor outcomes and ED care for Autistic people.

Another possible reason for this lack of applied research could be the exclusion of lived experience voices in research (Papastavrou Brooks et al. 2023; Gowen et al. 2019). Including these perspectives, if done ethically, is likely to lead to the generation of meaningful and translatable research (Nimbley et al. 2024) and can be done in a myriad of ways, including research conception, development, implementation, analysis and dissemination (Farr et al. 2021). While Autistic experiences have increasingly been explored in qualitative studies (Kinnaird et al. 2019a; Babb et al. 2022), and studies have reported the involvement of Autistic people with an ED in analysis (Brede et al. 2020; Nimbley et al. 2023a), there has been little involvement of the community in shaping what research gets done in the first place.

A recent study by Keller et al. (2024) reported research priorities as identified by Autistic people and people with ADHD who have disordered eating, which was broadly split between studies exploring how to improve outcomes (improving clinical services, psychoeducation and preventative medicine) and studies identifying causal mechanisms (identification of risk factors, and the role of autistic and ADHD neurocognitive profiles). Keller et al. (2024)'s study embodies an encouraging shift towards the inclusion of the lived experience communities in setting the research agenda and paves the way for future research to build and expand on their approach. For example, there remains scope to apply a more qualitative, accessible design that incorporates participant accounts and experiences. Furthermore, different ED presentations and mechanisms have been reported in Autistic populations compared to those with ADHD (Nickel et al. 2019), suggesting that there may be unique considerations and experiences in Autistic people worth exploring. Thus, there remains scope to apply more inclusive and accessible methodologies towards identifying research priorities in Autistic individuals with an ED.

Photovoice is a creative qualitative research approach that seeks to collect and reflect community experiences through the discussion of images (Wang & Burris 1994; Rose 2014). Photovoice aims to give voice to those with lived experience, engage participants through the construction and discussion of images, and reach the broader community with research findings (Saunders and Eaton 2018). A recent meta‐synthesis of Photovoice in autism research found the approach to be a useful and meaningful method to facilitate engagement with Autistic experiences through enhancing accessibility to research (Do et al. 2021). It has also been used to explore complex, sensitive topics in ED populations (e.g., Saunders et al. 2020), and its role in advancing eating disorder research has been advocated by researchers (Hower et al. 2022). The current study therefore sought to build on previous research, using inclusive and creative photovoice methodology to answer the following research question: *What are the research priorities for Autistic people with an ED?*

## 2 Methods

The pre‐registered protocol for the wider study can be found here: https://osf.io/sfgqm/.

### 2.1 Participants

Four groups took part in Photovoice sessions, ranging from two to five participants per group. Participants were eligible to take part in the study if they were over the age of 14, resided in the UK and were fluent in English. Participants were also required to self‐identify as Autistic or have a formal diagnosis of autism, as well as to have current or lifetime experience of an eating disorder. A total of 14 participants took part in the study, ranging between 19 and 48 years old. There was a relatively representative spread of genders across the sample, however most participants were white. All participants had received a formal diagnosis of autism, and a range of ED diagnoses and additional diagnoses were reported (see Table 1).

**Table 1:** Participant demographics (n = 14).

Age (Years)		Range	M (SD)
		19–48	31.07 (8.26)
Gender		***N***	**%**
	Female	8	57.1
	Male	3	21.4
	Nonbinary	2	14.3
	Transgender	1	7.1
Ethnicity			
	White	13	92.9
	Asian	1	7.1
Autism diagnosis			
	Clinical	14	100
	Self	0	0
ED diagnoses (self‐report)			
	Anorexia Nervosa	8	57.1
	Binge Eating Disorder	2	14.3
	ARFID	2	14.3
	EDNOS	1	7.1
	Not Disclosed	2	14.3
ED status			
	Current	10	71.4
	Recovered	1	7.1
	Unsure	3	21.4
Co‐occurring Diagnoses			
	Anxiety	9	64.3
	Depression	7	50
	ADHD	4	28.6
	Learning Disorder	4	28.6
	PTSD	2	14.3
	Complex PTSD	1	7.1

Abbreviations: ADHD, Attention Deficit Hyperactivity Disorder; ARFID, Avoidant/Restrictive Food Intake Disorder; EDNOS, Eating Disorder Not Otherwise Specified; M, mean; PTSD, Post Traumatic Stress Disorder; SD, standard deviation.

Participants were asked to complete self‐report measures of Autistic traits (AQ‐10, Allison et al. 2012) and eating disorder symptoms (Eating Disorder Diagnostic Scale—EDDS, Stice et al. 2000; Nine Item Avoidant and Restrictive Food Intake Disorder (ARFID) Screen—NIAS, Zickgraf and Ellis 2018—see Table 2). Self‐reported ED diagnosis matched the EDDS on four (44.44%) of eligible cases (*n* = 9). Of the remaining five cases, three self‐reported AN diagnoses and one OSFED diagnosis were assigned as High‐risk bulimic‐type, and one self‐reported BED diagnosis was assigned as BN.

**Table 2:** Descriptive statistics of completed self‐report measures.

ED diagnoses (n = 9, EDDS^a,b^)		N	**%**
	Anorexia Nervosa	3	33.3
	Bulimia Nervosa	1	11.1
	Binge Eating Disorder	1	11.1
	OSFED/EDNOS	0	0
	High risk bulimic‐type	4	44.4
NIAS^b^ score (*n* = 1)			Total score
			31
AQ score (*n* = 14)			M (SD)
			9.15 (0.98)

Abbreviations: ED = eating disorder, EDDS = Eating Disorder Diagnostic Schedule, EDNOS = Eating Disorder Not Otherwise Specified, NIAS = Nine Item Avoidant and Restrictive Food Intake Disorder (ARFID) Screen, OSFED = other specified feeding or eating disorder.

^a^Out of the total 14 participants, one participant reported a diagnosis of ARFID only which is not included as a diagnosis in the EDDS, two participants did not complete the EDDS, and two participants did not report their BMI so could not be included.

^b^Of the two participants who reported they have received a clinical diagnosis of ARFID, only one participant completed the NIAS.

### 2.2 Procedure

Adverts and information sheets were shared through the Eating Disorder and Autism Collaborative (EDAC: Duffy et al. 2024) mailing list, third sector organisations (SWAN, Scottish Autism) and from posts on social media platforms (e.g., X, Instagram). Interested participants were asked to contact the research team should they wish to take part, and eligibility was confirmed through email correspondence or by a short, online video chat. Participants were sent a link to an online survey platform (Qualtrics), where they were asked to provide consent and demographic information, including their age, gender (male/female/nonbinary/transgender/prefer not to say), ethnicity (white/black/mixed race/Asian/prefer not to say/other with open‐text response), as well information regarding co‐occurring conditions (clinical diagnoses, support needs) and ED status (current/recovered/unsure). Participants were then asked to complete self‐report measures of Autistic traits and eating disorder symptoms. These measures were included as indicators of sample characteristics and were not used for screening or exclusion purposes.

Workshops consisted of 5 weekly 1 h sessions for a total of four groups, taking place between May and July 2024. Workshops were run by a research team drawing on autism and ED research expertise, as well arts‐based and lived experience perspectives. Group workshops were chosen over individual workshops due to feedback from our collaborators within the Autistic and ED community, as well as previous studies supporting the motivating and supportive role that having participants and researchers with shared experience in the group can have on study participation (Bengtsson‐Tops and Svensson 2010; Pellicano et al. 2022). Three groups were held online, and one group took place in‐person. Both online and in‐person options were offered to participants to accommodate for communication preferences, and to offer alternatives in an effort to balance out previous studies highlighting both the positive impact on research engagement and participation that accessible online interviews can have (e.g., Realpe et al. 2023) and the possible limitations of online research in selection and sampling bias (Rubenstein and Furnier 2021).

Session 1 was an orientation and welcome session, outlining research purposes, building trust, establishing ground rules and how to approach taking the images. Session 2 and Session 3 covered two research questions, the latter of which explored research priorities for Autistic people with an ED. For each of these sessions, participants were asked to provide three images. Research questions were introduced at the end of the previous workshops, and participants were reminded again of the questions in a debrief email (e.g., at the end of Session 2, we discussed the research questions for Session 3 at the end and followed these up with an email going over the questions). Session 4 and 5 involved reviewing emerging themes from each of the research questions, which were developed between sessions by Autistic and non‐Autistic members of the research team Participants were sent the emerging themes before the session, and given the opportunity to provide feedback both during and after the workshop. To make the sessions more accessible, videos were shared of how to upload images and alternative text was provided for each of the images shared during the sessions. Participants were asked to share a description or narrative with their images, and discussion of the images could take place via the chat function or through their microphone. Image descriptions, chat messages and workshop recordings were transcribed and anonymised.

### 2.3 Data Analysis

Data from the images and anonymised transcripts of the sessions were analysed using a two‐step approach. Firstly, an analysis framework adapted from Wang and Hannes (2020) Photovoice Question Matrix was applied by two researchers (EN, KB) to Photovoice images looking at the production, content and meaning attributed to the images. Themes from the workshops and narratives were then identified using inductive, reflexive thematic analysis (Braun et al. 2023). Researchers familiarised themselves with the transcripts before conducting several roles of coding. Initial themes were developed at reviewed at this stage by a team of Autistic and non‐Autistic researchers, before themes were developed and finalised. In this way, multiple researchers and perspectives were employed to ensure a reflexive approach and to uphold Autistic voices through the analysis process. Due to the reflexive nature of the analysis, a data saturation approach was not employed, as reflexive approaches allow for constant potential for novel insights and interpretations (Braun and Clarke 2021). Instead, researchers were shaped by the adequacy of the emerging data in addressing research questions and mutually agreed when analyses were felt to have reached sufficient information power (Malterud et al. 2016). When applied, this led to the additional recruitment of a fourth group and the decision to end recruitment once these workshops had been analysed. This was done by the same two researchers, at which point an Autistic peer researcher (EM) provided feedback on emerging themes. Themes were then finalised by all three authors.

### 2.4 Researcher Reflexivity

It is important to reflect on the researcher's expertise and experience, and to acknowledge their positionality when approaching the research (Braun et al. 2023). EN is a neurotypical researcher who has worked extensively with the Autistic and ED community and brought her research and community experience to the workshops and to the analysis. Despite this, EN is aware of the limitations interpreting the data solely through a neurotypical lens and of the importance of being led by lived experience. KB's comprehensive experience working with Autistic people, combined with his own lived experience of Anorexia Nervosa, granted a degree of shared understanding which allowed him to effectively interact with participants during the workshops. However, KB recognises that as a researcher, his contribution is underpinned by a power differential which, together with being neurotypical, impacted the lens through which he understood these discussions and his subsequent analysis of them. EM is an Autistic peer researcher with ADHD and living experience of Anorexia Nervosa with a background of third sector and policy work in the field of autism and eating disorders. Being neurodivergent has facilitated connection, communication, and understanding with research participants. However, EM brings her own lived/living experiences to her research and does not represent the broader Autistic community.

## 3 Results

Five themes were identified that highlighted research priorities for Autistic people with an ED (see Table 3). These themes will be presented in the following section and will be supported by relevant images and quotes from Photovoice sessions. Key information on production (technologies) and content (visual elements and principles of design) from the image analysis will be presented below each image. From these themes, a range of preliminary research questions have been suggested in Table 4.

**Table 3:** Table of themes outlining research priorities.

Theme	Sub‐themes
1. Impact of early experiences	1a. Internalizing of socio‐cultural food and body image narratives
	1b. Generational cycles
2. Function of the ED	2a. ED as a regulatory strategy
	2b. ED as a social acceptance strategy
3. Barriers and facilitators to ED recovery	2a. Autistic traits as barriers
	2b. Autistic traits as facilitators
	2c. Help and harm of unravelling
4. Understanding and accommodating for complexity	4a. Co‐occurring conditions
	4b. Intersectionality
5. Changing research culture	5a. Inclusive and participatory research
	5b. Nonclinical support

**Table 4:** Possible research questions for future autism and ED research.

Theme	Possible research questions
Impact of early experiences	*How do Autistic children and adolescent learn rules around eating and food?*
	*What is the impact of internalizing body image ideals during childhood for Autistic people?*
	*How does the learning of rules around eating and food in childhood impact the development of an ED in Autistic people?*
	*How do Autistic people with an ED experience parenting?*
	*Explore the role of intergenerational cycles of EDs for Autistic people*.
Function of the ED	*How does disordered eating function as cognitive regulatory strategy for Autistic people?*
	*How does disordered eating function as sensory regulatory strategy for Autistic people?*
	*How does disordered eating function as social regulatory strategy for Autistic people?*
	*How does disordered eating function as a strategy to be accepted or to fit in for Autistic people?*
	*What is the role of social camouflaging in EDs in Autistic people?*
Barriers and facilitators to ED recovery	*How do Autistic characteristics, such as sensory differences, impact ED recovery?*
	*How can Autistic characteristics, such as routine and rigidity or intense interests, facilitate ED recovery?*
	*How do Autistic characteristics and ED behaviours interact?*
Understanding and accommodating for complexity	*Explore the role and impact of hormonal imbalances (e.g., testosterone) in Autistic experiences and presentations of EDs?*
	*How do GI conditions and difficulties impact Autistic experiences and presentations of EDs?*
	*How do autoimmune conditions impact Autistic experiences and presentations of EDs?*
	*What is the role of trauma in ED development for Autistic people?*
	*How does mental health difficulties impact ED experiences and treatment?*
	*How does ethnicity impact ED experiences and treatment for Autistic people?*
	*What are the experiences of Autistic males with an ED?*
	*What are the experiences of transgender Autistic people with an ED?*
	*What is the role and experience of gender diversity for Autistic people with an ED?*
Changing research culture	*How can we effectively use participatory research designs to coproduce evidence‐based training in ED services?*
	*Using participatory research designs, how effective are adjunct interventions (e.g., creative therapies) in supporting Autistic people with an ED?*
	*Using participatory research designs, how effective are community support strategies (e.g., peer support) in supporting Autistic people with an ED?*

### 3.1 Theme 1. Impact of Early Experiences

A leading research priority theme identified was understanding the impact of early environment on the Autistic child. This theme centred on exploring the importance of childhood and early development on ED outcomes in Autistic individuals: *“what happens when we are younger fixes so much as we [get] older” (Participant 9)*.

**1a. Internalizing Socio‐Cultural Food and Body Image Narratives**

A subtheme of this priority focused on understanding the Autistic experience of internalizing narratives around food and body image during childhood. This theme focuses on *how* these ideals are learnt from potentially autism‐specific differences in cognition (e.g., literal or black and white thinking) as opposed to *behavioural responses to* internalization (e.g., masking or camouflaging). Participants discussed the process through which messages around eating and body image are internalized:

*Participant 5: There was a lot of messages around [my body]. And I guess some of it was thinking about like, it's like do Autistic people hold messages and rules. I was taught that it was important… you know those messages would affect anybody. But it's like as an Autistic person who is learning to navigate the world through figuring out the rules*.

*Participant 1: A really powerful image and it sounds like it resonates with a lot of us*.

Participants felt that understanding this process and how it may lead to the development or maintenance of their ED should be a future research priority. In these discussions, participants wondered whether this was a unique Autistic experience, playing a more significant role in EDs for Autistic people than in non‐Autistic peers:

*Participant 2: The rules we tend to make or create in our minds and they become so like powerful, they're so important to us. It's like hard to justify like why I should have this type of food or this amount of food or this kind of brand of food. And then, I don't know, I just feel like the rules cannot be broken… it's that kind of black and white thinking, like, no, you cannot break it. Whereas I've heard experiences from those who have eating disorders not coming from that autistic kind of lived experiences. It's not as, as much, if that makes sense*.

**Image 1:**
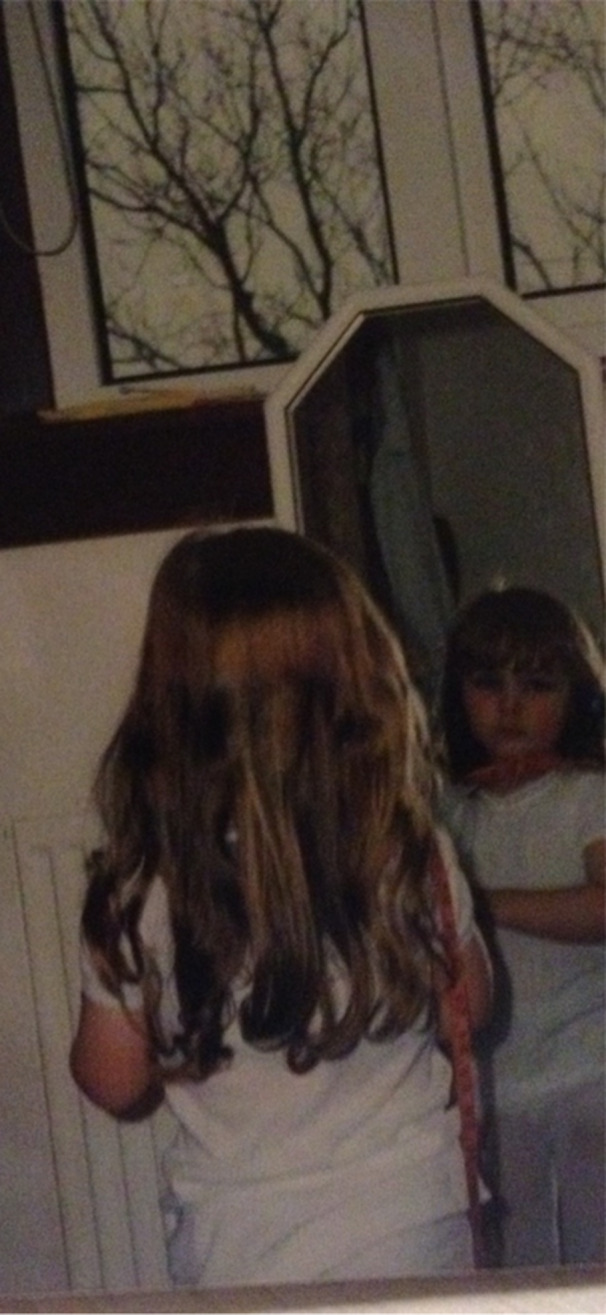
“What you say sticks”. *Production of the image:* Camera. *Content of the image*: Space is used to emphasise the small child and their reflection as the centre of interest.

Thus, participants felt that future research should prioritize understanding this process of internalization of socio‐cultural messages around food and body image narratives for Autistic people, and how this might be a unique pathway towards the development of eating disorders in this population. (Image 1)

**1b. Generational Cycles**

Another subtheme of discussions around the impact of early experiences focused on understanding generational cycles of EDs for an Autistic person. Participants reflected on the influence that childhood environments had on the development of their ED and how research should begin to explore this. Participants also discussed how important it was to break these cycles:

*Participant 4: How do we break the generational cycle of eating disorders? How do we sort of make that support so that the younger people that are coming up through life now are able to kind of realise, like, how can they have sort of a healthy relationship with food, what can we do to kind of stop that continual thing?*

**Image 2:**
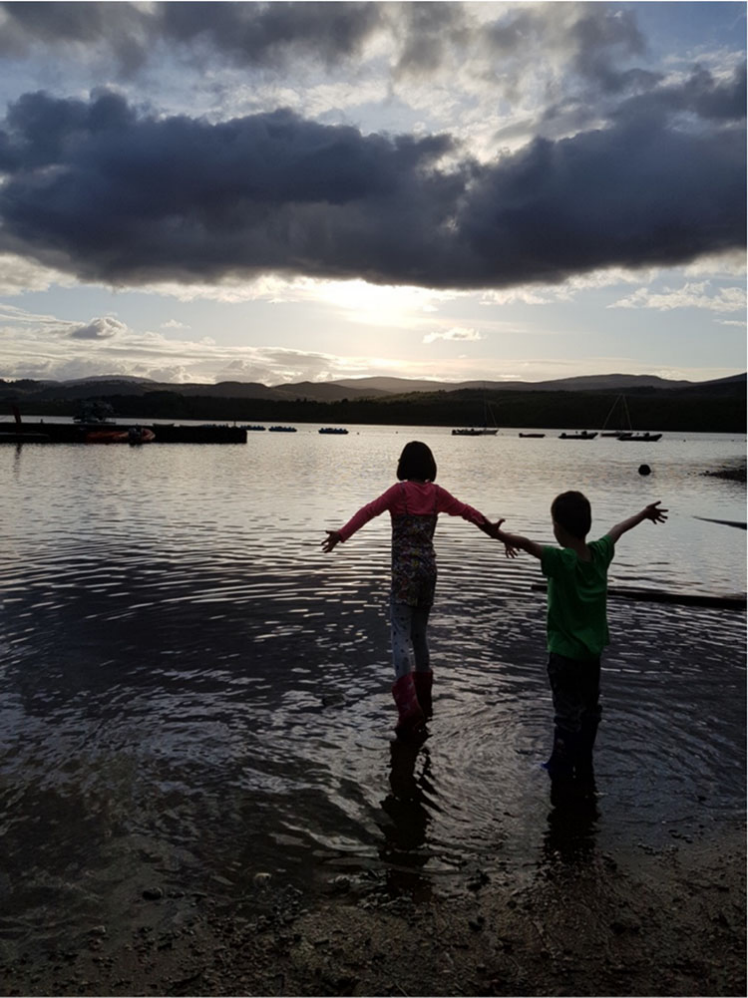
“Stopping”. *Production of the image*: Camera. *Content of the image:* Light and form are used to emphasise the children; line and tone are used for contrast between the dark water and light of the sun.

While this was discussed broadly across groups, several participants reflected specifically on their experience of approaching this as a parent:

*Participant 9: I guess for me that picture is about trying to break that cycle. It's about trying to create something for my children that is different. So, I guess that picture is one moment where I feel like I managed to do that. You know, one moment where I managed to put my own shit behind me* (Image 2).

### 3.2 Theme 2. Function of the ED

Across groups, participants discussed the need for research to explore the function of EDs in Autistic populations. Images and discussions centred around two leading mechanisms that participants felt required further research: the ED as a means of regulating sensory, emotional and cognitive factors (*1a. ED as a regulatory strategy*), and as a means of mimicking non‐Autistic traits to fit in with a neurotypical world (*1b. ED as a social acceptance strategy)*.

**2a. ED as a Regulatory Strategy**

For many participants, their ED was felt to regulate their responses to a range of stimuli and processes. This was discussed across a range of factors, including sensory sensitivities, as well as cognitive processing speed:

*Participant 11: I was trying to get across that food fuels my brain, [and] sometimes I don't want to think so fast, so I'll reduce the amount I'm eating to slow down my brain a bit*.

**Image 3:**
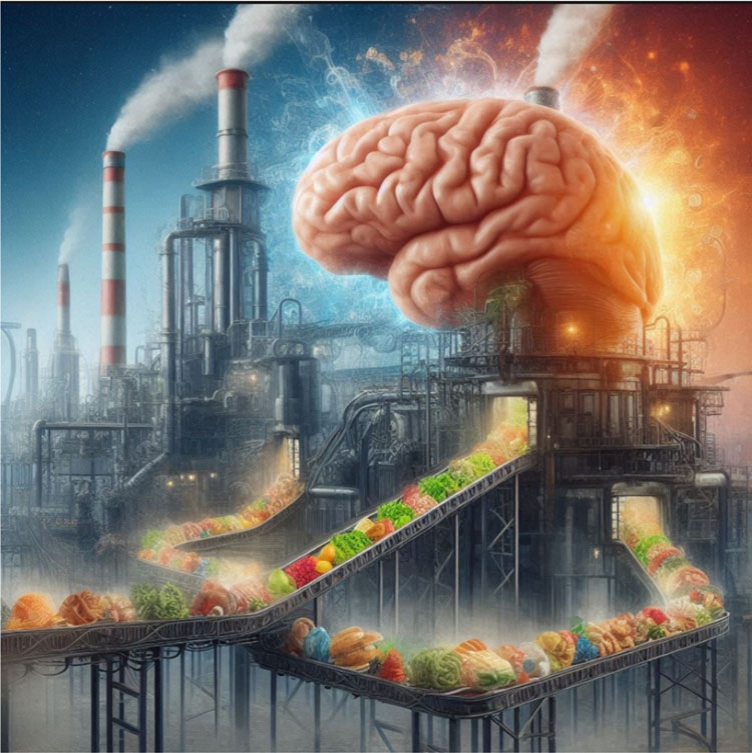
“Cognitive Plant”. *Production of the image*: Artificial Intelligence (AI). *Content of the image*: Line, colour and space combine to emphasise the brain as the centre of interest and repetition of beams, food items and towers.

*Participant 10: Interesting for future research that three people with autism find that they reduce [for] their brain activity. That was what my psychiatrist think is the whole cause of my ARFID, reducing food to cope* (Image 3).

**Image 4:**
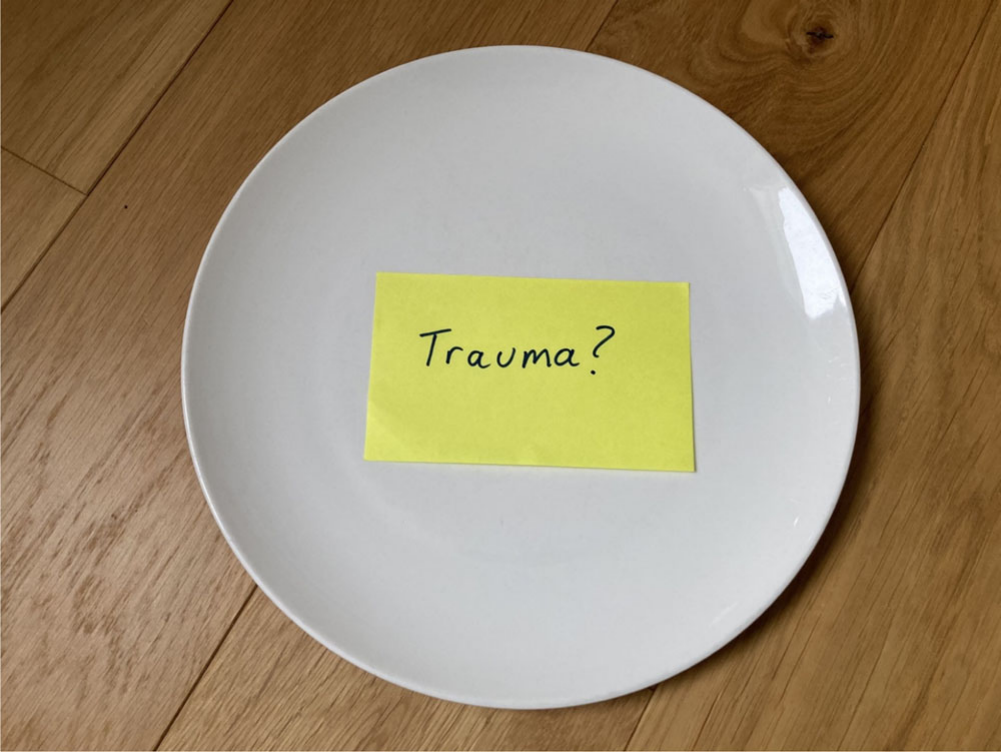
[Untitled]. *Production of the image:* Camera. *Content of the image*: Shape and tone are used to create contrast, emphasising the post‐it note.

This was felt to be a shared strategy across Autistic individuals, emphasising the importance of future research to understanding and support this. Another strategy discussed across participants was using the ED as a means of regulating their emotions, particularly distress:

*Participant 8: It was something that I sought comfort in…I was definitely seeking comfort and escapism from those kind of overwhelming feelings. I think that's probably the best way to put it* (Image 4).

For these participants, ED behaviours were a strategy to regulate emotions and to avoid feeling overwhelmed by these emotions, with another participant noting that their ED led them to get *“to a point where my body wasn't doing anything that wasn't absolutely necessary to keep me alive and emotions were something that I almost stopped experiencing” (Participant 7)*. While the impact of an ED on emotion processing and regulation is well documented across neurotypes, participants discussed *“problems with processing and expressing emotions, or alexithymia (Participant 7)*” as inherent and pre‐existing Autistic differences, highlighting the importance of researching Autistic experiences of EDs as an emotion regulatory strategy.

**2b. ED as a Social Acceptance Strategy**

The second subtheme reflects how participants felt that their ED was a means of attempting to fit in with or be accepted by a neurotypical world, and how future research should prioritise exploring Autistic experiences of this. Participants often discussed how they felt different when growing up and how these differences made them feel like they needed to repress themselves or mimic neurotypical traits to fit in. Participants discussed practicing facial expressions in the mirror or, specific to the ED, changing their body shape as a way of feeling accepted.

*Participant 5: I really idolized her in a way…And it was like, yeah, I think that added to it in itself of almost like, you know, trying to almost mimic that. Being like, if I was like her, I would fit in. If I was like this other popular person who also looks like this, I would fit in. [Being thin] just felt like it was taught everywhere, and it was a way of fitting in and being accepted. (see* Image 1, *What you say sticks)*.

This could start from different observations or environments, from individuals or from societal norms:

*Participant 8: And at the same time are trying to fit in and, and trying to sort of meet an ideal of what they think that, you know, you're trying to act like you think other people think should act, in the same time, you think ‘I should look like a normal person’, you know, especially when there's, you know, you feel the societal expectations on how you should look as well*.

Ultimately, participants felt conforming to neurotypical food and societal body ideals would lead to being accepted, and this process was felt to be central to their ED. Understanding how this develops and the impact that this can have on the Autistic individual was a shared research priority across groups.

### 3.3 Theme 3. Barriers and Facilitators to ED Recovery

The third theme identified as a research priority was understanding barriers and facilitators to ED recovery for Autistic people. Specifically, participants felt that there was need for a better awareness and understanding of how Autistic traits can interact with the ED, working to both prevent and promote recovery from their ED.

**3a. Barriers to Recovery**

A range of ways in which Autistic traits or characteristics could interrupt ED recovery were reported, such as differences in emotion processing and expressing emotions, as well as sensory sensitivities:

Participant 7: [Sensory experience of clothes] *makes being happy about all the things I can do difficult because I can't go out the house because I feel so uncomfortable in what I'm wearing. So then that freedom I'm supposed to have now isn't really there. And I think, you know, finding a way to help with that side of things and making that process easier would, would be really beneficial*.

**Image 5:**
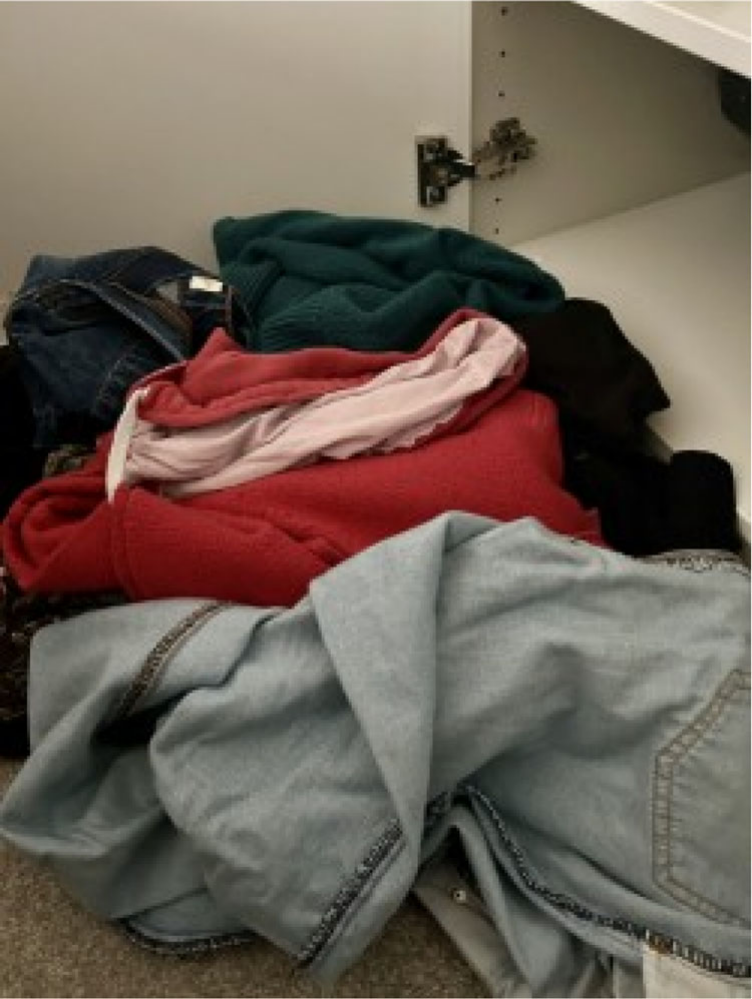
“Sensory Nightmare Pile Up”. *Production of the image*: Camera. *Content of the image*: Space and form is used to emphasise the bundle and to create asymmetrical content.

This was experienced both via sensitivities to clothes and internal bodily sensations, including temperature. Participants discussed how difficult and distressing it was to regulate their body temperature, with one participant reflected *that “is a whole other variable to a situation that I did not have to think about before (and it turns out I get very overwhelmed very fast when I'm too hot)” (Participant 6)*. Discussions around how internal body sensations, such as hunger and digestion, can impact the ED and recovery were echoed across groups. Participants suggested that future research should prioritise understanding the impact of sensory factors not just at a modality level, but also how different internal sensations are processed and integrated, and how these can be accommodated for in ED treatment:

*Participant 6: For me digestive stuff it isn't about, like, I mean, sometimes it is painful, but it isn't the pain situation of it. And it isn't like the unpredictability. It feels like textures on the inside and I can't get away from that. And like that's, that's the problem*. (Image 5)

**3b. Facilitators of Recovery**

Mirroring this, participants also reflected on how Autistic traits can be harnessed during ED recovery and how future research should prioritise understanding and facilitating this. This could take the form of intense interests being a motivator for recovery, as well as harnessing preference for routine. Many participants felt that this was something viewed in a negative way if looked at through an ED lens, but from an Autistic perspective, it was often felt to be something that could promote recovery if understood and accommodated for:

Participant 5: *I'm Autistic, I'm going to approach my recovery like I'm Autistic. I feel like being Autistic [can] sometimes be a blessing and a curse in terms of recovery and relapse and both whatever. But, like, that actually being routine really almost helped? I felt like if I'd had that support and continue with, to maintain that routine and that recovery… that like genuinely I would probably mostly nearly be recovered from my eating disorder*.

Participant 4: *Totally agree [Participant 5]! I found routine and reminders extremely helpful to avoid eating too much throughout the day*.

**Image 6:**
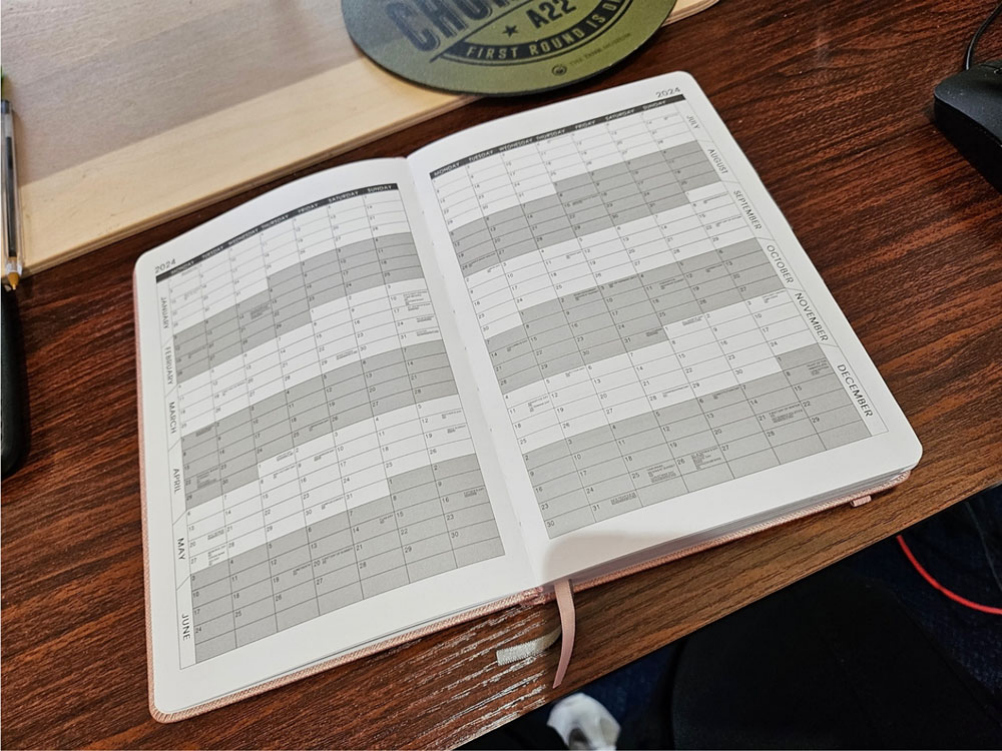
[Untitled]. *Production of the image*: Camera. *Content of the image*: Line and colour are used to convey symmetry, balance and repetition.

Of note, this was discussed across different ED presentations, including both restrictive and binging eating behaviours. Thus, the importance of understanding how Autistic traits, such as routine and intense interests, can promote recovery was felt to be a transdiagnostic research priority (Image 6).

**3c. Help and Harm of Unravelling**

**Image 7:**
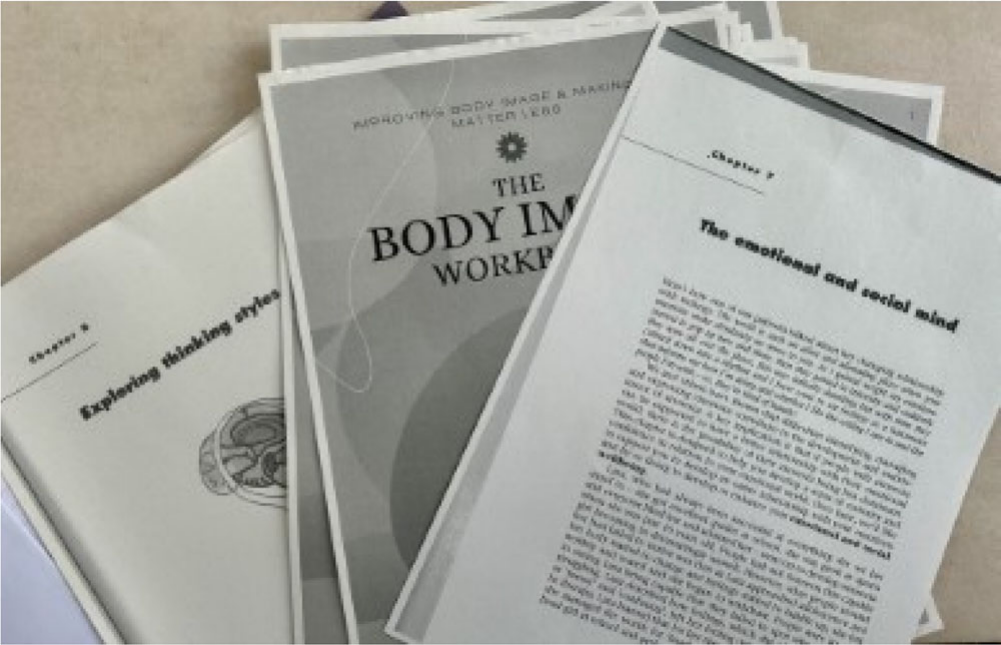
“I am Autistic, I am not my Anorexia”. *Production of the image:* Camera. *Content of the image*: Line and shape are used to create emphasis and symmetry.

Across these discussions, participants raised an important tension that may rise when seeking to ‘untangle’ Autistic traits from ED behaviours, with participants reflecting on both the help and the harm that this can cause. Of note, there was no consensus across participants as to how or even whether this should be approached by research (Image 7).

Participants who felt they had benefitted from an untangling approach discussed this in the context of working out what behaviours or thoughts were part of their ED or their illness and what parts of them were down to who they were as an Autistic person:

Participant 7: B*ecoming more flexible in my thinking this was not something I was going to be able to do as I naturally have a preference for routine and familiarity. Attempting to change this or challenge it in some way would have created even more distress to me and wouldn't be something I would be able to maintain. It was important for me to understand the difference between what anorexia was controlling and what was just my way of being*.

This was echoed by other participants, who discussed how this approach allowed them to notice how Autistic traits and ED behaviours may differentially influence their eating, with one participant noting that “*personally for me, it has actually been useful to try and separate those aspects of my life and impact that they have on my eating” (Participant 9)*. This untangling was not universally felt to be helpful, however. Some participants questioned this approach, discussing how they felt this was an often an impossible and potentially harmful task:

Participant 6: *Just to kind of reflect on the kind of figuring out thing like…I sometimes get really frustrated with people like now when people are kind of like ‘oh is that your autism or?’ and I'm like ‘I don't freaking know’ like I've only been me…I don't know how there was something that I can separate out and there's some things not and it's sometimes really frustrating*.

This sense of frustration was a leading reflection across those who found untangling to be unhelpful, with one participant saying that *“you can't really separate the two…100% we have to stop trying to separate and just have everything together and look at it as the whole person. I don't know why but it makes me so angry*” (Participant 4). A possible way to resolve this debate could be viewing it as an interaction, and, most importantly, adopting an autism‐affirming framework through which future care and research should approach untangling autism and EDs:

Participant 9: *I guess it's the thing of like, if, if it's useful for you to pick it apart, if it's useful for us to pick it apart, then having professional support to do that is really, really useful. But if someone else is trying to pick it apart to minimize something or diminish something or challenge us in a way that we don't find supportive or helpful, then maybe that's not a helpful route to go down*.

### 3.4 **Theme 4.** Understanding and Accommodating for Complexity

**Image 8:**
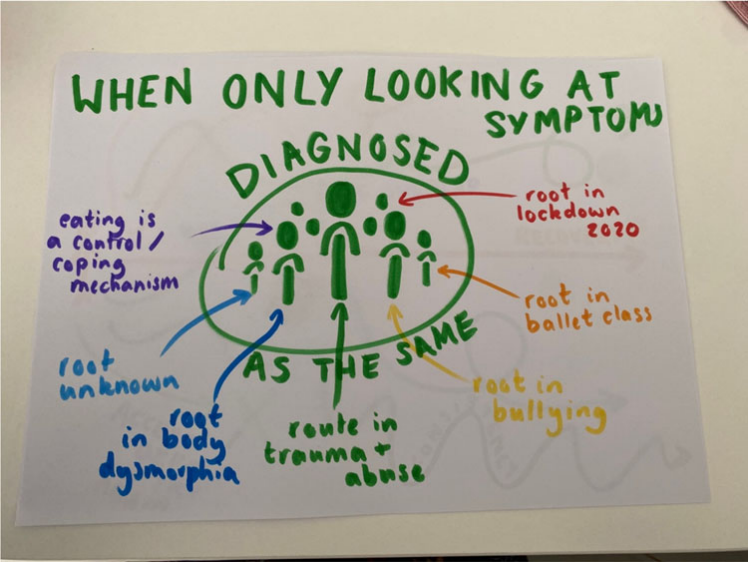
“One Category Fits All”. *Production of the image*: Drawing and camera. *Content of the image*: Use of space and symmetry emphasises the shared diagnosis, while colour is used to reflect possible variety in factors that can underlie the diagnosis.

Another leading theme across groups was the importance of understanding and accommodating for complexity. Participants felt that researching and understanding the complexity of the Autistic experience and its interaction with the ED will lead to improved outcomes:

*Participant 10: To treat someone, you do need to look at cause and I think if you took me and someone else with ARFID, then our treatments will be so incredibly different because of the different routes and you know, there are so many different reasons for I think like categorising people, not just by their symptoms but by, how this has come about it's going to be a lot more effective to treat them*. (Image 8)

Importantly, participants felt that consideration of both these elements – increasing awareness and incorporating into treatment approaches – were a notable research priority for Autistic people with an ED. Examples of this complexity were discussed across two broad sub‐themes: *Co‐occurring conditions* and *Intersectionality*.

**4a. Co‐occurring Conditions**

A universal experience across participants was the presence of co‐occurring conditions. This was often co‐occurring mental health conditions, such as posttraumatic stress disorder (PTSD) and anxiety. Participants also frequently discussed the presence of physical conditions and difficulties. These included hormonal imbalances, which were “*consistently missed both by GPs, by eating professionals by every other professional…and that is simply because they weren't aware (Participant 12)”*, and a range of gastrointestinal (GI) conditions including, Ehlers‐Dahlos Syndrome, celiac disease or other GI conditions:

Participant 6: *It's really common [in Autistic people] to have like digestive related problems…and then also if you have an eating disorder and then you try and eat in like quote un‐quote ‘typical ways’, you quite often have like all sorts of digestive chaos as well…It's like, so I said in hospital, like, ‘I feel better when I don't eat.’ And they thought I meant like somehow like mentally or something, I feel better. And they were just told me that I didn't. And that was just completely dismissed*.

For these participants, a lack of understanding of co‐occurring conditions had a hugely negative impact on the individual's experience of their ED and ED support. Central to this theme was the importance of increasing understanding of these co‐occurrences, using this understanding to inform ED service provision and ultimately develop more effective ED support. Importantly, it was felt that research should not only focus on understanding these physical or mental health conditions, but also on understanding the impact of these conditions on the Autistic person and how this may interact with their ED:

*Participant 12: think that a definite research priority that needs looked into is why are those co‐occurrences more likely in Autistic people and how does that link in with treating the whole person? I think that's often overlooked*.

**Image 9:**
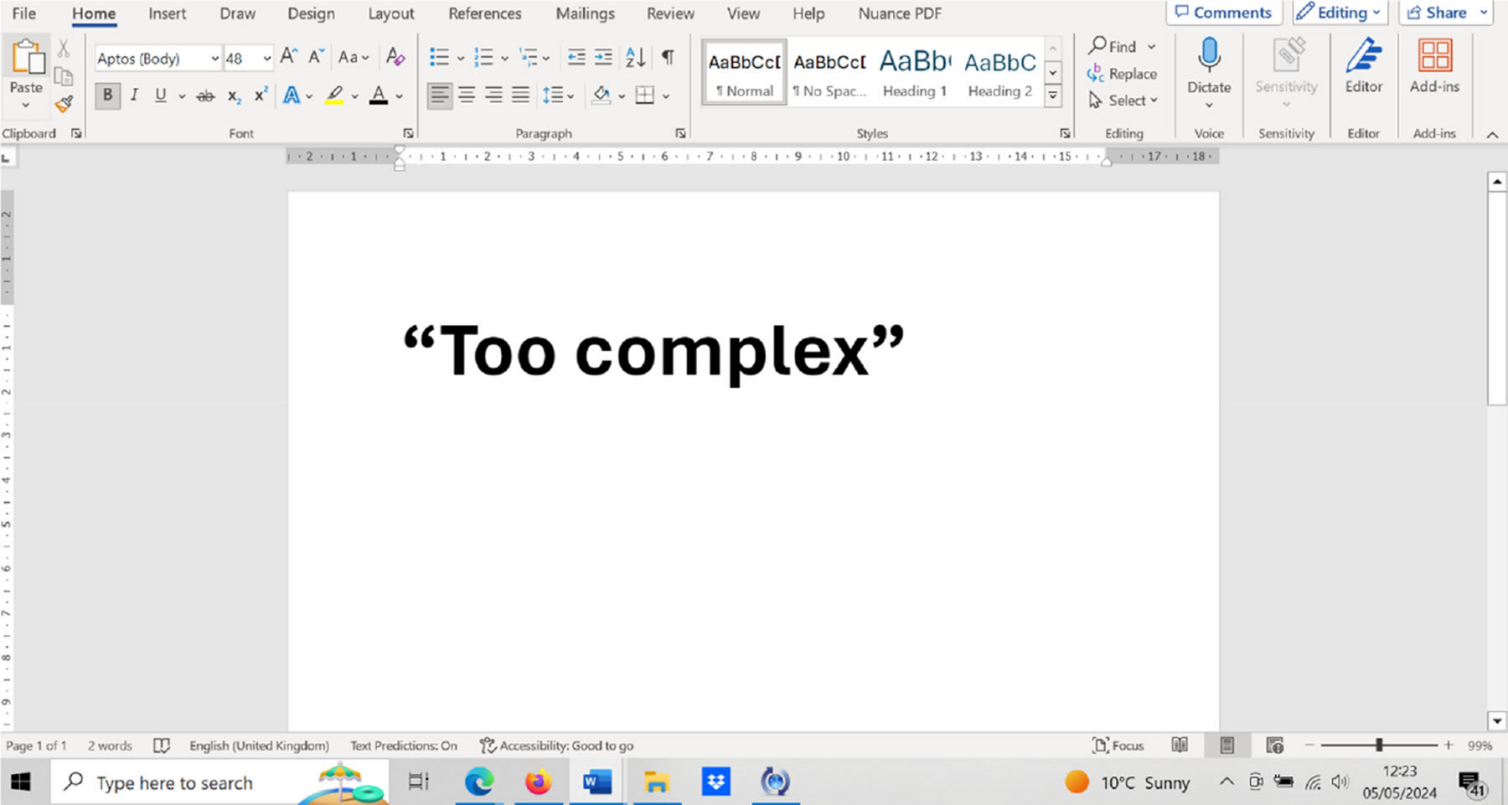
“Does anyone fit the mould?” *Production of the image: Computer screenshot. Content of the image: Space and tone are used to create a contrast between negative/white space and the words*.

*Participant 13: I think if they have the right education and understanding of what [co‐occurrences mean] and why they might be, it can just hopefully start those conversations and improve that awareness, which is really important* (Image 9).

**Image 10:**
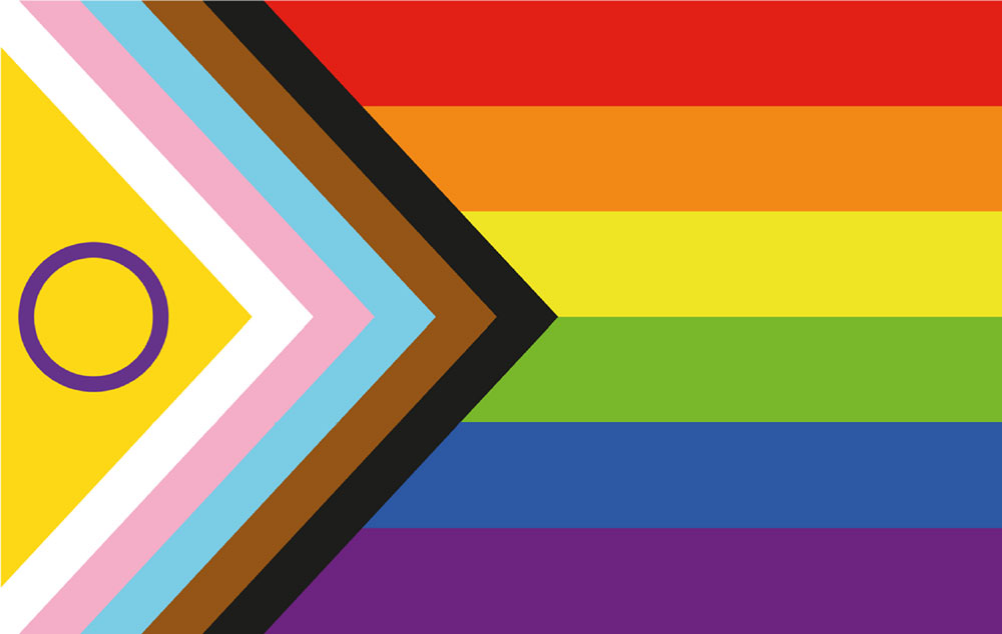
“Intersectionality”. *Production of the image:* Camera. *Content of the image*: Colour and line are used to emphasise different colours; form (of a flag) is used to emphasise unity.

**4b. Intersectionality (Image** 10)

Across groups, participants discussed the prevalence and impact of intersectionality within the context of autism and EDs. This was reported across several different domains; for example, different ethnicity and cultural norms around food were reported to play a significant role, and a lack of understanding within community and clinical support was noted as a barrier to effective care:

*Participant 2: Especially in my culture with within the‐ Indians and South Asians, there is that kind of culture of when you go to families and parents that love eating or overfeeding someone. And it's I guess their kind of hospitality or they just they want to kind of welcome and have, have people around and I suppose [they] use that food as a social aspect of gathering*.

*Participant 3: I just want to agree with that as well. I think that like, like you say, the sort of like different cultures aren't really like spoken about or acknowledged in like treatment and like teams*.

Gender was also discussed by several participants, who felt there are poor understanding of how transgender or male Autistic people experience their ED.

**Image 11:**
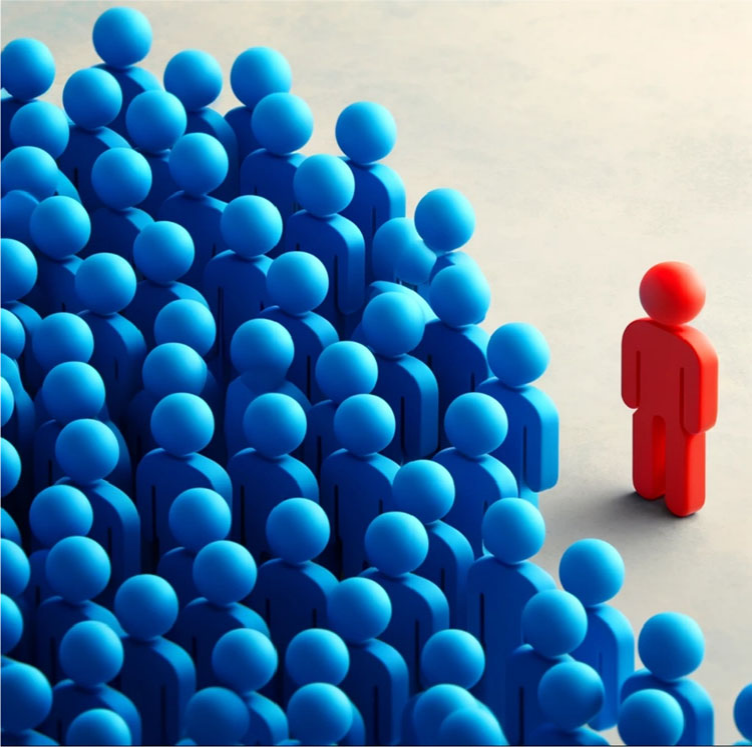
“Ginger Outcast”. *Production of the image:* Artificial Intelligence (AI). *Content of the image:* Colour and form are used to create contrast between the single red figure and the crowd of blue, and to centre interest on the single red figure.

*Participant 11: It reminds me of when I was at [services] they sent me to the nutritionist…but when I went, I don't think anyone told the nutritionist that I wasn't a girl. And they basically saw me and went I don't really know anything about male biology and then this is kind of specialised for girls, sorry* (Image 11).

This was often discussed in the context of male eating disorders, however several participants felt that this categorisation of genders may not always the best approach for future research exploring gender in Autistic people with an ED. One participant suggested that future research should seek to move away from neurotypical gender stereotypes, seeking to understand intersectionality as representative of the whole person:

*Participant 12: I think we over gender it sometimes. We talk about male eating disorders, and I've never seen someone talk about male eating disorders that isn't to do with gyms or body image of muscle dysphoria…and it's just not true. Well, it is true for some people but not for me, for a lot of people and especially for neurodivergent people, it's probably less true. We try to categorise things so much we lose sight of the person, and I think it's just the person we need to be looking at*.

### 3.5 **Theme 5.** Changing Research Culture

The final theme identified as a research priority was changing research culture. This was discussed across many different contexts, including prioritising research that will create an accessible and equitable culture (*5a. Inclusive and participatory research*) and improving preventative approaches within the community (*5b. Adjunct and community support*).

**5a. Inclusive and Participatory Research**

Another approach to improving clinical and research culture was felt to be creating a more welcoming and inclusive research culture. Many participants felt that they had previously been discriminated against or excluded from research and ED treatment and that transparency and trust should be promoted in future research:

*Participant 3: I'm hoping that, like, the training and priorities of treating people with autism and eating disorders is welcoming everyone's point of view and it's not like discriminating against anyone… I guess what I'm saying is that I think that it's really important to not judge anyone and not discriminate against anyone and welcome everyone with more open arms because from my experience sometimes that's not happened*.

**Image 12:**
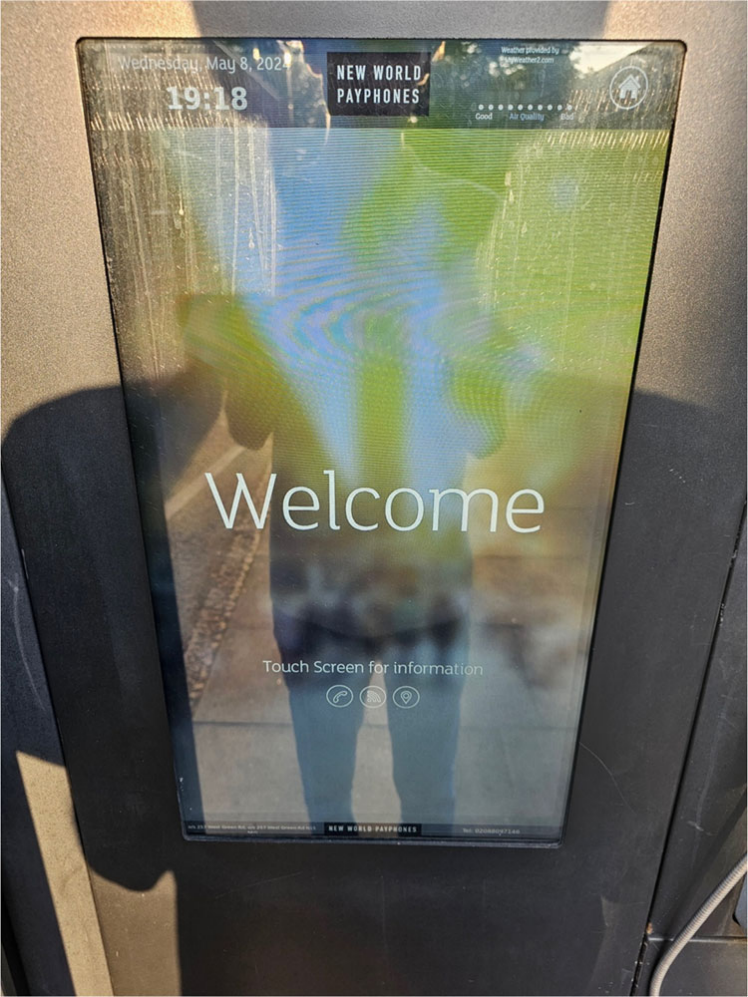
“Hello, Welcome”. *Production of the image*: Camera. *Content of the image*: Shape and line is used to imply balance and to create a contrast that emphasises the screen.

Participants also reflected on a lack of knowledge about Autistic presentations of EDs, strongly advocating for future research to prioritize informing improved education and training within ED services. Participants also emphasized the importance of having Autistic voices at the heart of this:

*Participant 1: I think if practitioners in this area are more informed and or more individuals are upskilled to work with people with autism and associated eating disorders that this could potentially be life changing for some. I also think that as part of developing any education for professionals, it would be important for them to engage with those who've got lived experience* (image 12).

**5b. Adjunct and Community Support**

The final subtheme identified was the importance of enhanced and autism‐informed adjunct or community support for Autistic people with an ED. Across these different contexts, participants fundamentally emphasized that future research should prioritize developing interventions and measuring the efficacy of these interventions that are more aligned to Autistic needs. For example, several participants discussed creative therapies and the importance of research‐driven understandings into how these therapies can work *“as a distraction from eating disordered thoughts and other negative thoughts, and also I find it easier to eat” (Participant 9)*. Participants also discussed this from a preventative perspective, reflecting on the distressing and unique environment of inpatient services and how important it was for future research to inform improved community care out with clinical settings:

*Participant 14:* I *think for me, real recovery happens in the real world, and I think a way to prevent that is really where the research should be, or at least making community support robust and looking at ways for it to be adapted for people with ASD. I think a research priority is just trying to get the right support in the community for people and how to make that better, how to keep people at home, not let is escalate*.

**Image 13:**
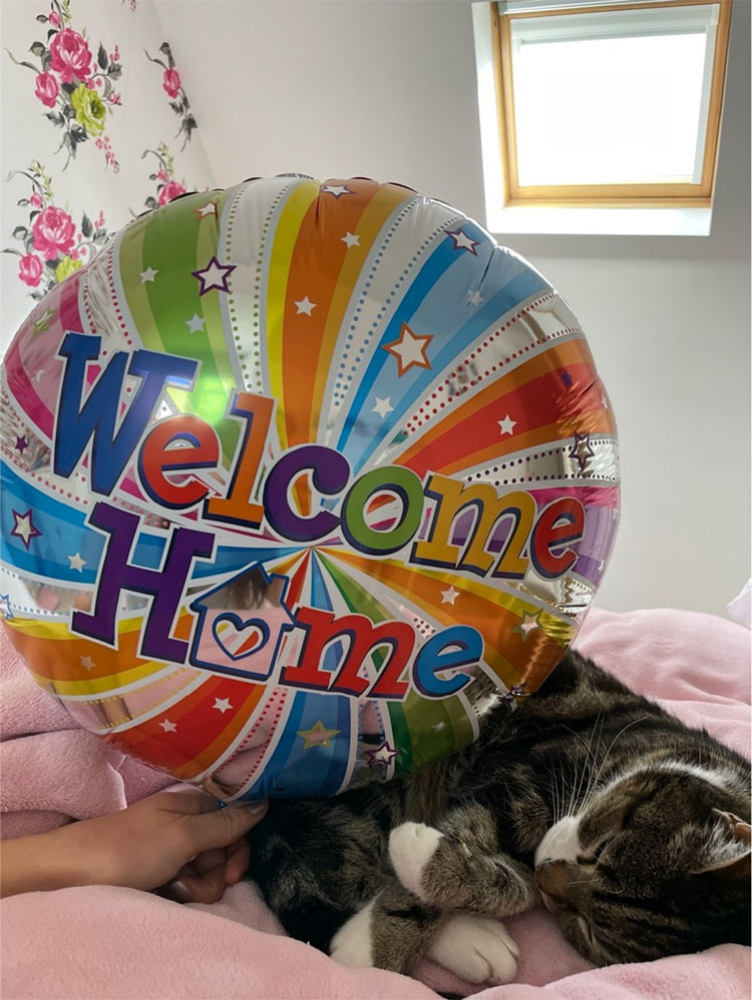
“I just wanted to be at home with my cat”. *Production of the image*: Camera. *Content of the image:* Colour and form is used to create emphasis on the closeness of the cat and the home sign.

By prioritizing research that will develop and improve community support, participants felt that the need for inpatient care would be reduced and that they can begin to recover in the “*real world*”. (Image 13)

## 4 Discussion

The current study sought to explore research priorities in Autistic people with an ED using an inclusive and creative Photovoice methodology. Five areas of future research were identified: (1) The impact of early experiences; (2) Function of the ED; (3) Barriers and facilitators to ED recovery; (4) Understanding and accommodating for complexity; and (5) Changing research culture. Only one study to our knowledge has sought to identify research priorities for neurodivergent individuals with an eating disorder (Keller et al. 2024). There were shared or similar priorities identified, such as a need for neurodiverse‐affirming care and training within services as well as the importance of identifying mechanisms. However, the qualitative, creative approach adopted in the current study and the focus on Autistic experiences allowed for the exploration of more in‐depth priorities focusing on a range of self, social and societal processes.

Autistic participants with an ED identified understanding the impact of early experiences on the development of their ED as a leading research priority. The majority of autism and ED research has been conducted on adults or young adults, with a dearth of studies recruiting child or adolescent samples (e.g., Karlsson et al. 2013), perhaps partially due to the mis‐ or under‐diagnosis of autism in females (Lai et al. 2015; Gesi et al. 2021) leading to autism being raised for the first time during their presentation to ED services (Babb et al. 2022). A range of different multi‐disciplinary methodologies could be drawn upon in future research to explore the nature and impact of early experiences, such as other arts‐based designs or mixed‐method approaches seeking to contextualise outcomes within these experiences. Longitudinal studies could also be a promising methodology for exploring the impact of early experiences. Only a handful of longitudinal studies have been conducted in the field, tentatively establishing an association between childhood Autistic traits and adolescent disordered eating (Solmi et al. 2021; Carter Leno et al. 2022) and notable long‐term ED and psychosocial outcomes (Leppanen et al. 2022; Nielsen et al. 2015; 2022). Such approaches will help researchers understand the impact of early experiences, including factors implicated in the current study such as trauma, narratives around food and body image, and cognitive processes implicated in the learning or internalizing of rules.

Another leading research priority for Autistic people with an ED was understanding the function of the ED, discussed across two different strategies. The first consisted of using the ED as a regulatory strategy for sensory, emotional and cognitive processing. While studies have tentatively explored how sensory sensitivities and interoception may lead to disordered eating (Adams et al. 2022; Nimbley et al. 2022), there is little to no prior investigation into the regulatory function of eating behaviours on emotion and cognitive factors that are commonly implicated in autism, alexithymia (Kinnaird et al. 2019b), ‘cognitive rigidity’ or preference for sameness (Brede et al. 2020) and information processing (Williams et al. 2015). Future research could therefore seek to understand how ED behaviors, such as restricting or binging, may be employed as a strategy to regulate cognitive or emotional processing. Furthermore, while the focus of the current study was on Autistic experiences, future research may consider exploring these strategies in Autistic and non‐Autistic individuals with ADHD given their common co‐occurrence (Hours et al. 2022); for example, whether food restriction may similarly be used as a means to slow down cognitive processing.

The second strategy warranting future research was using the ED to fit in or to feel accepted by others, in line with recent evidence to implicate social camouflaging as a leading factor in EDs for Autistic people (Bradley et al. 2024). Research exploring this strategy should be wary of confusion around terminology, with the terms camouflaging and masking used interchangeably (e.g., Cage et al. 2018; Cage and Troxell‐Whitman 2019) and inconsistent terms used between the research and Autistic community. In line with the Camouflaging Autistic Traits Questionnaire (CAT‐Q; Hull et al. 2019), participants in the current study discussed mimicking non‐Autistic behaviors to fit in (assimilation) and differs from masking strategies which seek to hide or repress Autistic traits (Radulski 2022). Future research could explore the role of social camouflaging strategies on ED behaviours and could inform improved support for Autistic people with an ED by highlighting social camouflaging as an important consideration during treatment.

Another suggested avenue for future research was exploring how Autistic traits or characteristics can influence the ED, either as a barrier or as a facilitator to ED recovery. While a number of studies have sought to establish autism‐specific mechanisms (e.g., Brede et al. 2020; Adams et al. 2024), to our knowledge no study to date has looked at the relationship between these variables and ED treatment outcomes. Given that Autistic people have been found to have poorer ED treatment outcomes and experiences (Nimbley et al. 2025), future research could seek to understand the impact of these traits and how they can be harnessed to promote recovery. For example, intuitive and flexible eating is a typical goal of ED treatment, however, the current study suggests that such an approach may not be suitable for Autistic populations where preference for consistency and routine or differences in processing of internal sensations such as hunger and satiety are baseline characteristics (American Psychiatric Association, D. S. M. T. F., & American Psychiatric Association, D. S 2013; Petrolini et al. 2023; Adams et al. 2022). Additionally, intense interests, or monotropic or hyper‐focused tendencies (Murray et al. 2005), have been implicated as a motivating factor in other areas, such as creativity and artistic hobbies discussed in the current paper (de Schipper et al. 2016), and could be harnessed as a strength during ED treatment. An important caveat to this line of research—seeking to unravel what is autism and what is the ED – is to acknowledge the harm and confusion that seeking to separate the two can cause. Instead, it may be best to view it as an interaction, looking to understand how the Autistic characteristics interact with the ED behaviours and vice versa. Research findings could be incorporated into current interventions that are being adapted and developed for Autistic people with an ED, such as the PEACE Pathway (Pathway for Eating disorder and Autism developed from Clinical Experience; https://peacepathway.org/). There is emerging and encouraging evidence to support the implementation and efficacy of the PEACE Pathway (Tchanturia et al. 2021; Li et al. 2024; Paphiti et al. 2025), and future research exploring how Autistic traits may help or hinder ED recovery can further contribute to similar pathways seeking to improve ED treatment outcomes for Autistic people.

Future research should also seek to explore the “complex” and heterogenous nature of autism, seeking to improve awareness and understandings of how these complexities can influence ED presentations. For example, EDs are still poorly understood in Autistic males (Murray et al. 2017) and transgender and nonbinary individuals (Heiden‐Rootes et al. 2023). Mental health difficulties, such as anxiety, depression and PTSD, are elevated in Autistic people (Lai et al. 2019; Haruvi‐Lamdan et al. 2020; Rumball et al. 2021), as are a range of physical conditions, including Ehler's Danlos Syndrome (Casanova et al. 2020), GI issues (Madra et al. 2020) and hormonal imbalances (Dangmann 2023). Little research, however, has been conducted into how these complexities impact ED experiences and outcomes. A real concern of this lack of research is diagnostic overshadowing, whereby symptoms of the underlying conditions may be misattributed to the ED or vice versa, leading to poor care and outcomes (Hallyburton 2022). Future research should draw on Autistic experiences of these complexities and how they may interact with ED symptoms, seeking to inform psychoeducation and awareness within ED services and to improve ED outcomes and experiences for Autistic people.

Autistic participants with an ED also made suggestions for future research should include participatory research approaches and seek to develop autism‐informed support. Advocating for the inclusion of Autistic voices in research is in line with strengths‐based, neuro‐affirming approaches (Singer 2017), which recognize neurodivergent individuals (with divergent brain function and cognition from what is considered to be ‘typical’, as in autism) as part of a broad spectrum of human diversity, shifting away from medicalized and ableist approaches that seek to ‘cure’ autism. The importance of such approaches has been highlighted across clinical (Chapman and Botha 2023; Lerner et al. 2023) and research (Dwyer 2022; Nimbley et al. 2023b) settings and thus it is recommended that future autism and ED research be rooted in neurodiversity‐affirming principles. An example of this in research is participatory research, or coproduction, which is research that involves lived/living experience perspectives across all stages of the research process; what gets done, how it gets done and how it gets shared (Cornwall and Jewkes 1995; Farr et al. 2021). One avenue for future participatory research could be the refinement or development of autism‐specific ED measures. Currently, there is a stark lack of ED measures that have been validated in Autistic populations, with concerns raised that current measures are failing to capture Autistic experiences (Longhurst et al. 2024). This is possibly highlighted in the current study through the inclusion of the EDDS, which was used to confirm disordered eating (compared to Autistic eating behaviors) and to demonstrate diversity in ED representation. While the fact that the study included participants at any stage of their ED could partially explain this, the EDDS was still only successful in diagnosing under half of the sample, suggesting that it may not be the most accurate reflection of Autistic experiences of an ED. Participatory design could also be utilized in support services to improve outcomes for Autistic individuals within the community. A suggestion from the current study was to embed Autistic people with an ED within teams delivering education and training to ED services, ensuring that strategies are guided by experience and that those going through services can benefit from peer support.

The current study has several strengths. The accessible nature of Photovoice methods and the flexibility between online and in‐person workshops meant that a broad range of Autistic experiences were included. This accessibility was particularly important in light of feedback from the Autistic community surrounding difficulties with prioritizing or deciding which area was the most important. A range of ED diagnoses and genders across participants were also reported and is a notable improvement from previous research biases towards females with anorexia nervosa (Flores et al. 2022; Mandy and Tchanturia 2015). However, the current study was biased towards White participants, raising concerns about representativeness of ethnicity. Furthermore, while efforts were made to make the study inclusive of and accessible to a broad range of participants (e.g., accommodating different communication preferences and processing times), study design was not suitable for non‐verbal participants, raising further concerns about sample representativeness. Furthermore, there remains scope to map the research priorities identified onto existing research, given that priorities were aligned with participant experiences and not necessarily with the current state of the literature.

The current study used inclusive, arts‐based methods to identify research priorities for Autistic people with an ED. Several directions for future research have been suggested and it is hoped that these inspire novel, interdisciplinary studies, deepening our understanding of EDs in Autistic people. Importantly, these priorities have been identified by the Autistic community, informing the investigation of meaningful research topics. It is hoped that future research will continue to involve collaboration between the Autistic community and researchers, with the community continuing to inform what research is done, and researchers working to ensure that this study is done in a rigorous and ethical manner. Through this approach, emerging research will be guided towards areas that reflect the realities of the Autistic community and can serve as an evidence‐base to be translated into improving the lives of Autistic people with an ED.

## Ethics Statement

Ethical approval was obtained from the School of Health in Social Science Research Ethics Committee at the University of Edinburgh (CLPS046).

## Consent

All participants provided informed consent to take part in the study.

## Conflicts of Interest

The authors declare no conflicts of interest.

## Data Availability

The data that support the findings of this study are available on request from the corresponding author. The data are not publicly available due to privacy or ethical restrictions.
